# Heavy metal enrichments in rural environment soils, pollution dynamics, source identification, and the role of subsurface mineralizations

**DOI:** 10.1038/s41598-025-22281-y

**Published:** 2025-10-14

**Authors:** Gökhan Demirela, Mustafa Haydar Terzi, Mehmet Barış Durgun

**Affiliations:** 1 Department of Geological Engineering, Aksaray University, Aksaray, 68100 Türkiye; 2 General Directorate of Mineral Research and Exploration (MTA), Ankara, 06530 Türkiye

**Keywords:** Anthropogenic, Geogenic, Soil pollution, Hydrothermal processes, Weathering, Lateral-vertical investigation, Environmental sciences, Environmental monitoring, Geochemistry

## Abstract

This study employed a multifaceted analytical approach, incorporating spatial distribution mapping, integrated pollution indexes, multivariate statistical methods, and geochemical comparative analyses to investigate the geochemical characteristics of heavy metals (HMs), determine the distribution of HMs, assess soil contamination levels, and elucidate the origins of HMs enrichment in soils. Notable concentrations of HMs in 719 soil samples were recorded, with levels reaching up to 33 ppm for arsenic, 114 ppm for cobalt, 746 ppm for copper, 2384 ppm for nickel, 53 ppm for lead, 128 ppm for vanadium, and up to 290 ppm for zinc. Various pollution indexes, including the degree of contamination (C_d_), modified degree of contamination (mC_d_), ecological soil pollution index (ESPI), and pollution load index (PLI), were calculated to delineate the levels of contamination. The index calculation results of soil samples revealed that 3.3%, 0.7%, 0.3%, and 0.6% of the samples were contaminated with HMs on the basis of the C_d_, mC_d_, ESPI, and PLI, respectively. The results of the correlation analysis demonstrated consistent and strong positive relationships between the pollution indexes, with a correlation coefficient greater than 84.4% at a significance level of 0.01. These findings also provided valuable insights for environmental risk assessment. The present study emphasized that anthropogenic sources are linked primarily to agricultural practices and former mining activities, while geogenic sources are primarily associated with weathering, oxidation, and hydrothermal processes occurring in local rocks and subsurface mineralizations.

## Introduction

Heavy metals (HMs) are major environmental contaminants due to their toxic characteristics. Today, HMs pollution in soil is becoming an increasingly serious environmental problem^1–7^. Soils act as a sink and source for various contaminants, especially HMs, including arsenic, nickel, and lead, which are notable inorganic contaminants in soils^7^. Their persistence poses a challenge for soil remediation^8^. In addition, their bioaccumulation in water and the food chain is influenced by the geological and environmental cycles. As a result, human health and the environment can be seriously affected^9–13^. Therefore, it is essential to monitor soil pollution carefully for the sake of the environment and human health. During the monitoring process, various methods can be employed, including soil sampling, determining HMs content, creating spatial distribution maps, calculating pollution indexes, performing statistical analyses, and conducting geochemical evaluations.

Soil sampling is the initial stage of the pollution evaluation process. Various studies have reported different depths for soil sampling, including 0–5 cm^14,15^, 5 cm^13^, 0–10 cm^16,17^, 0–15 cm^18,19^, 15 cm^13^, 0–20 cm^8,20^, 0–25 cm^21,22^, 0–30 cm^12,23–25^, 0–40 cm^26,27^, 0–50 cm^28^, and 60 cm^13^. The variation in depth of sampling depends upon the objectives of the study or on the different levels of the soil profiles. The B horizon in the soil profile is a sink of HMs and is the best site for sampling^25,27,29,30^. In the second stage, after preparing the soil samples for analysis, the HMs contents are determined via various analytical methods such as AAS^1,13,18,21^, ICP-AES/OES^14,16,31^, ICP-MS^1–4,22–25,29^, and ED/WD-XRF^1–4,12,13,27,28^. When selecting an appropriate analytical method for a study, it is important to consider features such as sensitivity, repeatability, and performance of the instrument. These factors can greatly impact the accuracy and reliability of the results obtained. The obtained geochemical data are used to generate the anomaly maps. These maps remain a crucial tool for comprehending the spatial distribution, variability, and average, as well as above-average, enrichment values related to HMs from the standpoint of geological and environmental sciences. The anomalous enrichments depicted on the geochemical distribution maps may be attributed to anthropogenic pollution arising from the clustering of residents, farming, industry, and mining in particular areas. An alternative possible geogenic source is that these distributions could be associated with the enrichments resulting from the weathering process of rocks and/or the presence of ore deposits, whether known or unknown^3,4,27,32–35^. Nowadays, various pollution indexes are employed for evaluating the degree to which the soil is contaminated and enriched with HMs and/or whether such levels jeopardize the ecological balance. The degree of contamination (C_d_)^36^, modified degree of contamination (mC_d_)^37^, ecological soil pollution index (ESPI)^12^, and pollution load index (PLI)^38^ are some examples of soil pollution indexes. These indexes are calculation and ecological classification systems that combine numerous HMs into a single score by utilizing the geochemical characteristics of the soil^12^. Multivariate statistical analysis, such as correlation, principal component (PCA), and hierarchical cluster (HCA) analyses frequently provide more reliable insight into the geochemical processes that oversee the coexistence of HMs and determine the source of HMs. In addition, realistic classification and interpretation of geogenic and anthropogenic sources of contamination necessitate certain lithological and lithogeochemical correlations. To classify potential sources of HMs enrichments in soils geochemically, it is necessary to compare soil geochemical data with standard geological materials, particularly those of local rocks.

The Geyve district (Sakarya, Northwest Türkiye) plays a significant role in Türkiye’s fresh fruit and vegetable production, functioning as one of the primary warehouse centers for surrounding cities, particularly Istanbul, Kocaeli, and Ankara^39^. According to the most recent data from 2023, the district has a population of 51,676, with nearly 7,000 families deriving their livelihood from agricultural activities. The increase in agricultural activities on a daily basis has given rise to a concomitant necessity for the establishment of new agricultural regions, mostly under forest cover. The region’s favorable climate allows for the cultivation of a wide range of agricultural products, with the exception of citrus fruits. The plain lands situated on the right and left banks of the Sakarya River are highly conducive to agricultural activities, with a diverse range of crops, including vegetables, fruits, and industrial products. The primary agricultural practice on the hillside lands is viticulture, with olive, cherry, tomato, and sunflower cultivation also being common. In the arid and mountainous regions, the predominant agricultural activity is grain farming. In recent years, the cultivation of walnuts, cherries, and sour cherries has increased^40^. The increase in agricultural activities has been accompanied by a corresponding increase in the use of pesticides and chemical fertilizers. Approximately 100,000 l of pesticides, including insecticides, fungicides, herbicides, acaricides, and rodenticides, are utilized annually. Additionally, 4,851,725 tons of chemical fertilizers, comprising a range of substances such as ammonium sulfate, magnesium nitrate, urea, potassium nitrate, potassium sulfate, compound fertilizers, and mixtures containing zinc and calcium nitrate, are employed on an annual basis^40^.

The soils in the Geyve region are predominantly developed over the Abant Formation, which is characterized by varying blocks of granite, serpentinite, quartzite, limestone, and conglomerate and typically interbedded with sedimentary sequences. Previous studies have investigated the level of HMs accumulation and potential sources of contamination of the soils and sediments of Sakarya and its surroundings, but most of these studies analyzed only a few soil samples from large regions and/or focused on very small regions. Moreover, these studies did not account for the potential influence of the links between soil geochemistry and local lithology on the interpretation of contamination sources^14,16,31,41–43^. Some studies did not consider potential anthropogenic sources of contamination and their environmental impact and only assessed HMs content in soils and sediments to explore mineral deposits in areas adjacent to the study area (e.g., the Kirpiyen and Örencik regions)^44,45^.

**Fig. 1:**
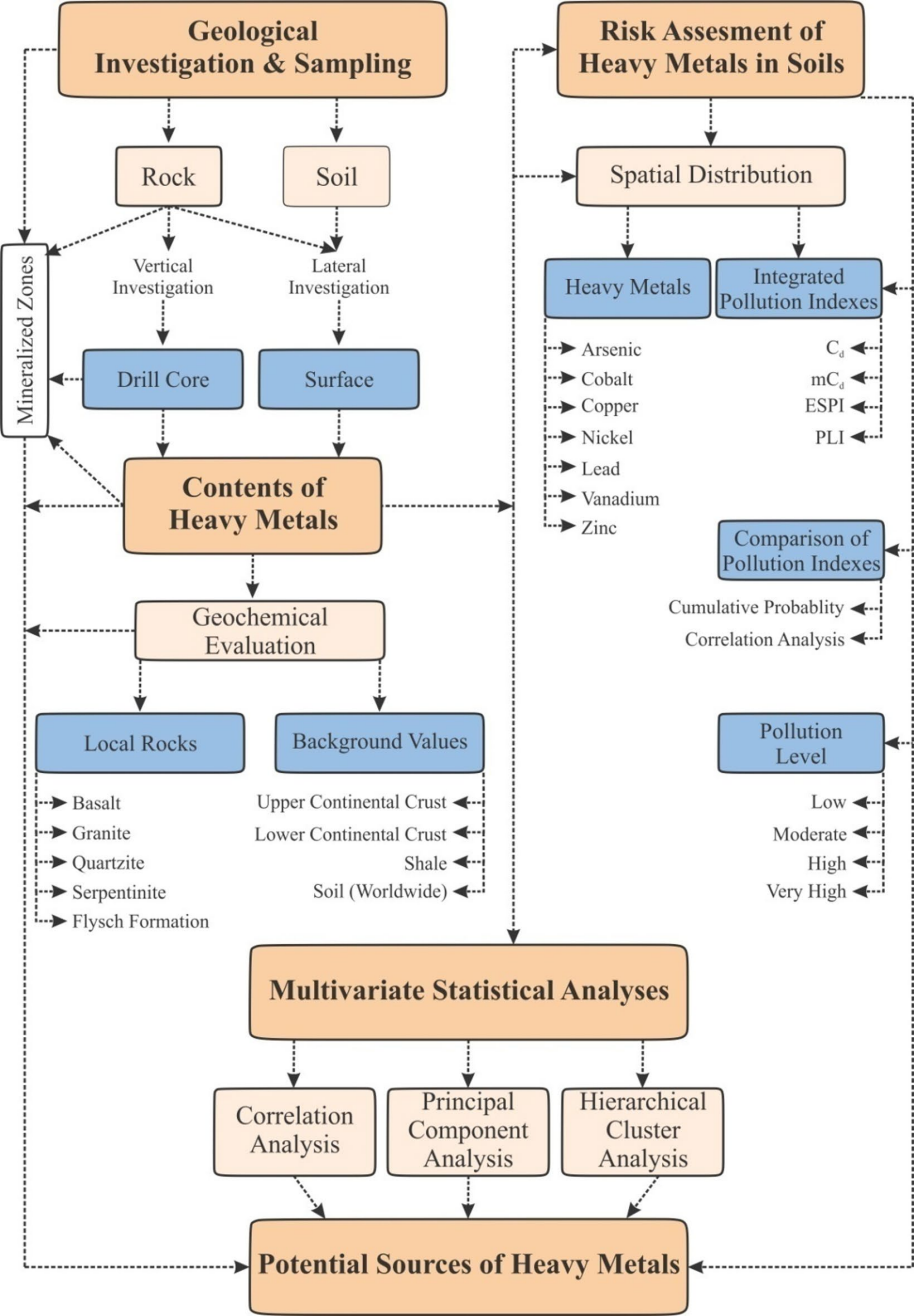
The general framework of the research methods.

This study aims to assess soil contamination levels and potential sources of HMs enrichment from both geogenic and anthropogenic perspectives. Additionally, it investigates the impact of two-dimensional (lateral) and three-dimensional (vertical) variables on soil pollution when distributing pollution sources in soils. The soil, surface rock, and drillcore samples in the case study area, located in the northeast part of the Geyve district (southwest of Sakarya), were subjected to geochemical analysis for evaluation. To ensure consistency, various evaluation methods were employed, including the creation of spatial distribution maps, calculation of four integrated pollution indexes, multivariate statistical analyses, and geochemical comparisons. This study contributes to the investigation of the geochemical characteristics of HMs, determines their distribution, assesses soil contamination levels, and elucidates the origins of HMs enrichment in soils. Furthermore, this study provides valuable insights into the assessment of environmental risks posed by HMs in soils through various pathways and is critically important for the planning of new agricultural and settlement areas. The general framework of the research methods used in this study is presented in Fig. 1.

## Materials and methods

### Study area

**Fig. 2:**
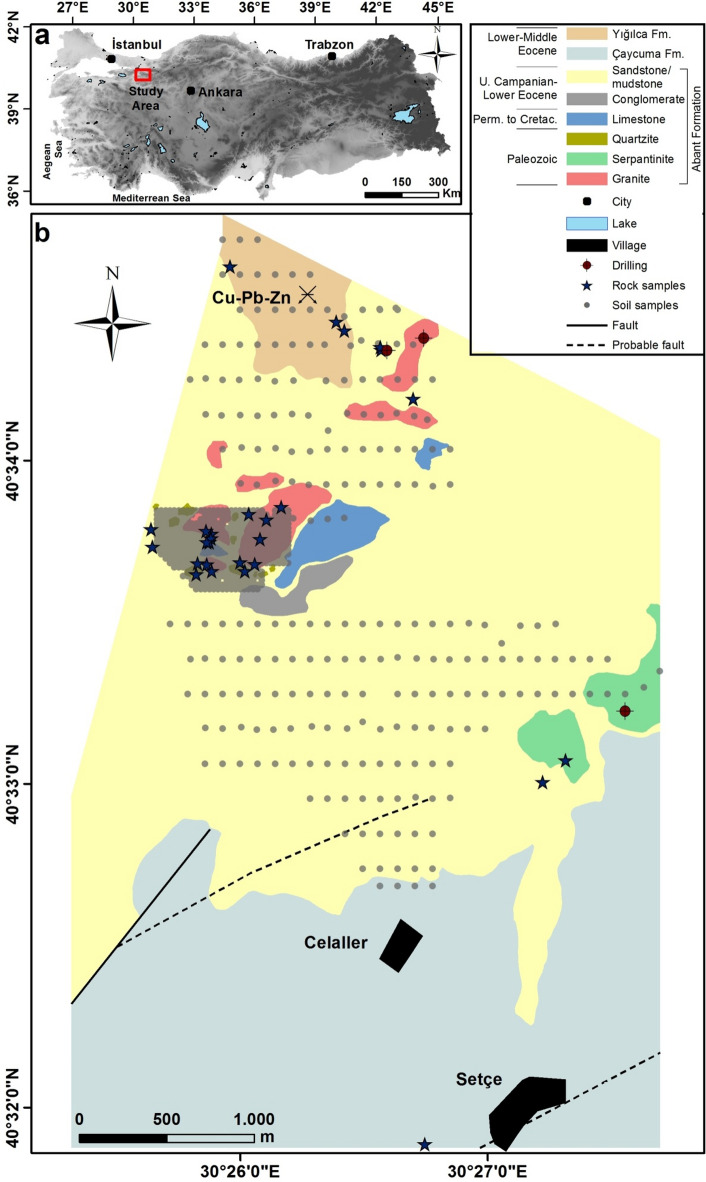
Location (a), geological and sampling maps (b) of the study area^57,58^ (The figure was generated by ArcGIS Pro 3.4. https://www.esri.com/en-us/arcgis/products/arcgis-pro/overview).

The study area is situated 12 kilometers northeast of the Geyve district in Sakarya city. It is bounded by the latitude range of 40°34’40’’ to 40°31’52’’ N and the longitude range of 30°25’09’’ to 30°27’41’’ E. The study area covers approximately 14 km^2^ on the Adapazarı G24c3 map (sheet 1/25000) (Fig. 2a). It is geographically and geologically situated in the western part of the Black Sea Mountains^46^ and the Pontides^47^, respectively. In the study area, the Armutlu-Almacik zone encompasses the Pamukova metamorphites and Abant complex (formation), whereas the Istanbul zone comprises the Çaycuma and Yığılca formations^48^.

The Pamukova metamorphites represent the basement rocks of the study area. However, they are not observed as outcrops in the study area. These rocks include different types of clastic, carbonate, and volcanic rocks, with traces of low-grade metamorphism^49^. These rocks are intruded by granitic intrusions^50^. The age of the granitic unit has been interpreted as Pre-Permian^51,52^ and Paleozoic^50,53,54^. The Late Campanian-Early Eocene Abant formation, which has an angular unconformity, overlies older rock units^45,55^. The Abant formation is a mixture of various rocks, including sandstone, siltstone, and mudstone. Their structure is quite complex: the sedimentary sequence consists of terrestrial and shallow marine formations with occasional platform carbonates of different ages and olistostrome characteristics, together with conglomerate, pelagic mudstone, serpentinite, quartzite, and granite blocks (Fig. 2b). The Çaycuma formation unconformably overlies the Abant formation and comprises sandstone, siltstone, and claystone in an alternating sequence (Fig. 2b). The fossil content indicates a depositional age in the Lower-Middle Eocene. The Yığılca formation, which unconformably overlies the Çaycuma formation, comprises mainly andesite, basalt, tuff, agglomerate, and volcanogenic sandstone (Fig. 2b). Its age is estimated to be Lower to Middle Eocene, as evidenced by the occasional occurrence of nummulite fossiliferous marl interlayers within the unit^45,48^. All rock units are stratigraphically unconformably covered by recent sediments of Quaternary age.

### Sampling and analytical procedures

The soils in the study area predominantly have dense vegetation, low to moderate slops, moderate to well-developed slops, and are characterized by gray, yellow, brown, and black colors. The thickness of the soil profile ranges from a few centimeter to 3 m. Soil samples were collected from 719 sampling sites via a handheld GPS device during the summer of 2021 (Fig. 2b). At each site, the soil profile corresponding to the B-horizon was identified, and samples were taken from an average depth of 30 cm. Approximately one third of the total soil samples (230 samples) were obtained throughout the study area at equidistant intervals of 100 m x 200 m, with a homogenous distribution of sampling points. The remaining 489 soil samples were collected in the central part of the study area at intervals of 25 m x 25 m. Various procedures, including labeling, drying, sieving, and pulverizing, were applied to the soil samples. Thirty surface rock samples were collected from five different groups of local rocks that are exposed in the study area. In addition, 651 drillcore samples were collected from three drillholes (S-1, S-2, and S-3) at a total depth of 931 m. Geochemical analyses were conducted by the laboratory of the General Directorate of Mineral Research and Exploration (MTA), a national government-owned mineral exploration agency that has been operating in Türkiye since 1935^56^. Seven HMs (As, Co, Cu, Ni, Pb, V, and Zn) were analyzed in the soil and surface rock samples, and six HMs (As, Co, Cu, Ni, Pb, and Zn) were analyzed in the drillcore samples by using Thermo Scientific iCAP 7000Plus Series-Inductively Coupled Plasma-Optical Emission Spectrometry (ICP-OES). The samples were digested using a modified aqua regia solution, which has the advantages of being consistent, economical, and rapid. The procedures for dissolution and ICP-OES analysis followed the TS ISO 14869-1 and SM 3120 B standards, respectively. To ensure high analytical reliability, a comprehensive QA/QC protocol was implemented throughout the study. This included the use of duplicate samples to assess analytical precision, blank samples to detect potential contamination, and certified reference materials to verify measurement accuracy. Laboratory equipment was meticulously cleaned between analyses to minimize the risk of cross-contamination. HM concentrations were cross-checked against accredited geological standards, and the relative percent difference between duplicate and original samples was calculated, with values below 5% considered within acceptable limits. The report identified the following detection limits: 3 ppm for As and Cu, 5 ppm for Co, Ni, Pb, and V, and 2 ppm for Zn. The samples that fell below the detection limit were divided in half and were included in the subsequent calculations and evaluations.

### Integrated pollution indexes

This study employed four integrated pollution indexes to assess the levels of HMs pollution risk in the soil samples. (i) C_d_; (ii) mC_d_; (iii) ESPI; and (iv) PLI. The methods used to calculate and classify the ecological risk of the soils based on the integrated pollution indexes in this study are presented in Table 1. These integrated pollution indexes have been widely used for evaluating the level of HMs pollution in soil and sediments^6,12,15,17–19,25,28–30,36–38,57–63^. Hakanson’s^36^ C_d_ provides an objective assessment of the contamination of HMs by classifying them into four categories ranging from low to very high. The contamination factor (C^i^_f_), an initial intermediate component of C_d_, is calculated by dividing the average content of an HM by its pre-industrial reference value^36^. The sum of the C^i^_f_ values obtained for each HM gives the C_d_ value (Table 1). Abrahim and Parker^37^ modified the C_d_ value to mC_d_. The level of contamination is determined by dividing the sum of the C^i^_f_ values of all the HMs by the number of HMs analyzed. The C_d_ is graded into seven classes, ranging from zero to very high in mC_d_ (Table 1). Yılmazer and Terzi^12^ formulated an AHP-based ESPI to determine the pollution risk in soils. ESPI has four classes, ranging from nil to high, which are used to categorize pollution risk in soil (Table 1). The pollution risk levels, which are based on the contamination factor (CF), are assessed via the PLI^38^. The CFs are calculated for each of the HMs, multiplied by each other, and then square-rooted at level n (number of CFs). The PLI evaluates pollution levels in two categories: unpolluted (< 1) and polluted (> 1) (Table 1)^38,59–63^. A background/reference value is used in the calculation of the integrated pollution indexes. This study used the shale mean value, which represents the uppermost level of the Earth’s crust and does not show any anthropogenic inputs^25,64–66^.

### Statistical analysis

Multivariate statistical analyses, including correlation, PCA, and HCA, were used to reveal the associations between the HMs and to elucidate the factors controlling geochemical processes in the soils of the study area^59–63^. Descriptive statistics, correlation analysis, PCA, and HCA were conducted via SPSS version 24. To establish the links between HMs, correlation analysis was performed at a significant level of 0.01. Principal components (PCs) with eigenvalues greater than 1 were obtained from the PCA to identify possible sources of HMs contamination in the soils. The varimax rotation method with Kaiser normalization was applied in the analysis. To assess the similarity of HMs in soils, R-mode HCA was carried out with Ward’s clustering method on the basis of Euclidean distance. The HMs contents were standardized with Z-scores to ensure equal influence for each HM before analysis. Furthermore, the spatial distribution of HMs and the results of the integrated pollution indexes were presented in maps generated via the ordinary kriging method with ArcGIS Pro 3.4.

**Table 1:** Calculation procedures and their risk classification of integrated pollution indexes.

Index	C_d_		mC_d_	
Equation	C_d_ = ΣC^i^_f_		mC_d_ = ΣC^i^_f_ / n	
	C^i^_f_ = (C^i^_0__−1_/C^i^_n_)		C^i^_f_ : contamination factor	
	C^i^_f_ : contamination factor		n = number of analysed HMs	
	C^i^_0__−1_: the mean content of the HM		i = ith HM (or pollutant)	
	C^i^_n_: the preindustrial reference value of the HM			

	Range	Category class	Range	Category class
Classification	C_d_ < 8	Low degree of cont.	mC_d_ < 1.5	Nil to very low degree of cont.
	8 ≤ C_d_ < 16	Moderate degree of cont.	1.5 ≤ mC_d_ < 2	Low degree of cont.
	16 ≤ C_d_ < 32	Considerable degree of cont.	2 ≤ mC_d_< 4	Moderate degree of cont.
	C_d_ ≥ 32	Very high degree of cont.	4 ≤ mC_d_< 8	High degree of cont.
			8 ≤ mC_d_< 16	Very high degree of cont.
			16 ≤ mC_d_< 32	Extremely high degree of cont.
			mC_d_ ≥ 32	Ultra high degree of cont.
References	^36^		^37^	

Index	ESPI		PLI	
Equation	ESPI = ∑W_m_ * P_fi_		PLI = ^n^√(CF_1_ x CF_2_ x CF_3_ x … x CF_n_)	
	P_fi_ = (M_i_/ S_vm_) * (M_i_/ (∑M_n_)		CF: contamination factor = C_metal_ / C_basevalue_	
	W_m_: the weight value for element M; the weight values of the HMs were used as 1 in this study		n: number of contamination factors	
	P_fi_ : the pollution factor for the alternative i			
	M_i_: the value of the element M for ith alternative			
	S_vm_: the standard value for the element M			
	M_n_: the sum of the values of element M			
	n: the number of elements			
	m: the any element			

	Range	Category class	Range	Category class
Classification	< 1	No pollution	PLI < 1	Unpollution
	1–3	Low pollution risk	PLI > 1	Pollution
	3–5	Medium pollution risk		
	> 5	High pollution risk		
References	^12^		^38^	

## Results

### Spatial distribution of HMs

**Fig. 3:**
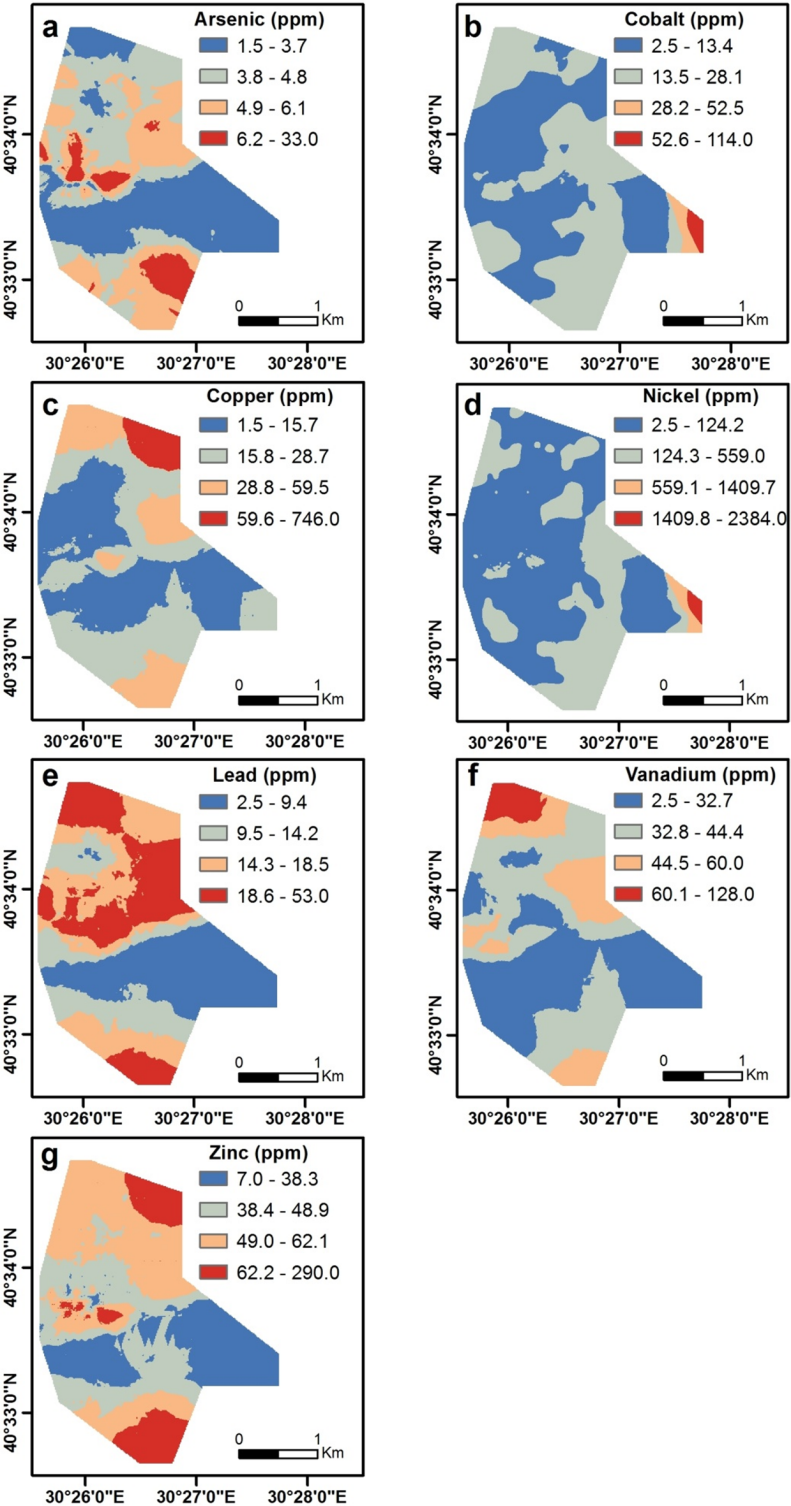
Spatial distribution maps for arsenic (a), cobalt (b), copper (c), nickel (d), lead (e), vanadium (f), and zinc (g).

The HMs contents of soil samples ranged from 1.5 to 33 ppm for As, 2.5 to 114 ppm for Co, 1.5 to 746 ppm for Cu, 2.5 to 2384 ppm for Ni, 2.5 to 53 ppm for Pb, 2.5 to 128 ppm for V, and 7 to 290 ppm for Zn (Table 2). The spatial distribution of the HMs is presented in Fig. 3. Arsenic contents show the increase towards the central, northeastern, and southwestern parts of the study area. The highest levels of arsenic (samples 350, 377, and 348) were detected in the central zones, corresponding to the flysch formations and granite areas (Fig. 3a). Sample 2 has the highest content of cobalt and is situated in the southeast of the study area, covered with serpentinites. The other samples (456, 337, 308, 480, and 331) with high cobalt levels are located where flysch formations and granite are prevalent (Fig. 3b). Elevated levels of copper are observed in the northern parts of the study area. The sample taken from the soil in the flysch formations, sample 632, has the highest content of copper. Samples 626 and 627, which are found in basalt outcrops, contain 87 and 105 ppm copper, respectively (Fig. 3c). The highest nickel content of 2384 ppm in the study area is located in the southeastern region, which corresponds with the serpentinite block in the flysch formations. The additional samples (514, 632, and 496) possessing elevated nickel levels are obtained from the flysch formations. The distributions of nickel are predominantly consistent with the cobalt pattern (Fig. 3b and d). The highest contents of lead are present in samples 65, 350, 348, 320, and 389. Lead contents increase in the central, northeastern, and southwestern sectors of the study area, which mostly agrees with the arsenic pattern (Fig. 3a and e). Vanadium levels are the most elevated in the northwestern parts of the study area (Fig. 3f). Four soil samples (622, 623, 627, and 628) containing the highest vanadium values were taken from soils on the basalt outcrops. Figure 3g demonstrates that high zinc contents are present in the central-western, northeastern, and southwestern parts of the study area. The sample with the highest zinc level (290 ppm) is in the soil of the flysch formations. In addition, samples (398, 320, and 388) taken from soils in granitic outcrops also contain high levels of zinc.

**Table 2:** Descriptive statistics for the geochemical results of the soil samples.

Descriptive value	As	Co	Cu	Ni	Pb	V	Zn
	ppm	ppm	ppm	ppm	ppm	ppm	ppm
Minimum	1.5	2.5	1.5	2.5	2.5	2.5	7.0
Maximum	33.0	114.0	746.0	2384.0	53.0	128.0	290.0
Mean	5.0	11.6	16.7	67.6	17.3	39.0	50.3
Standard Deviation	2.9	8.1	29.8	115.2	7.7	14.1	19.5
Skewness	2.7	3.4	20.6	11.9	0.5	0.8	3.0
Kurtosis	17.0	34.8	501.9	228.0	1.1	2.8	31.4
N	719						

### Geochemical evaluation

**Fig. 4:**
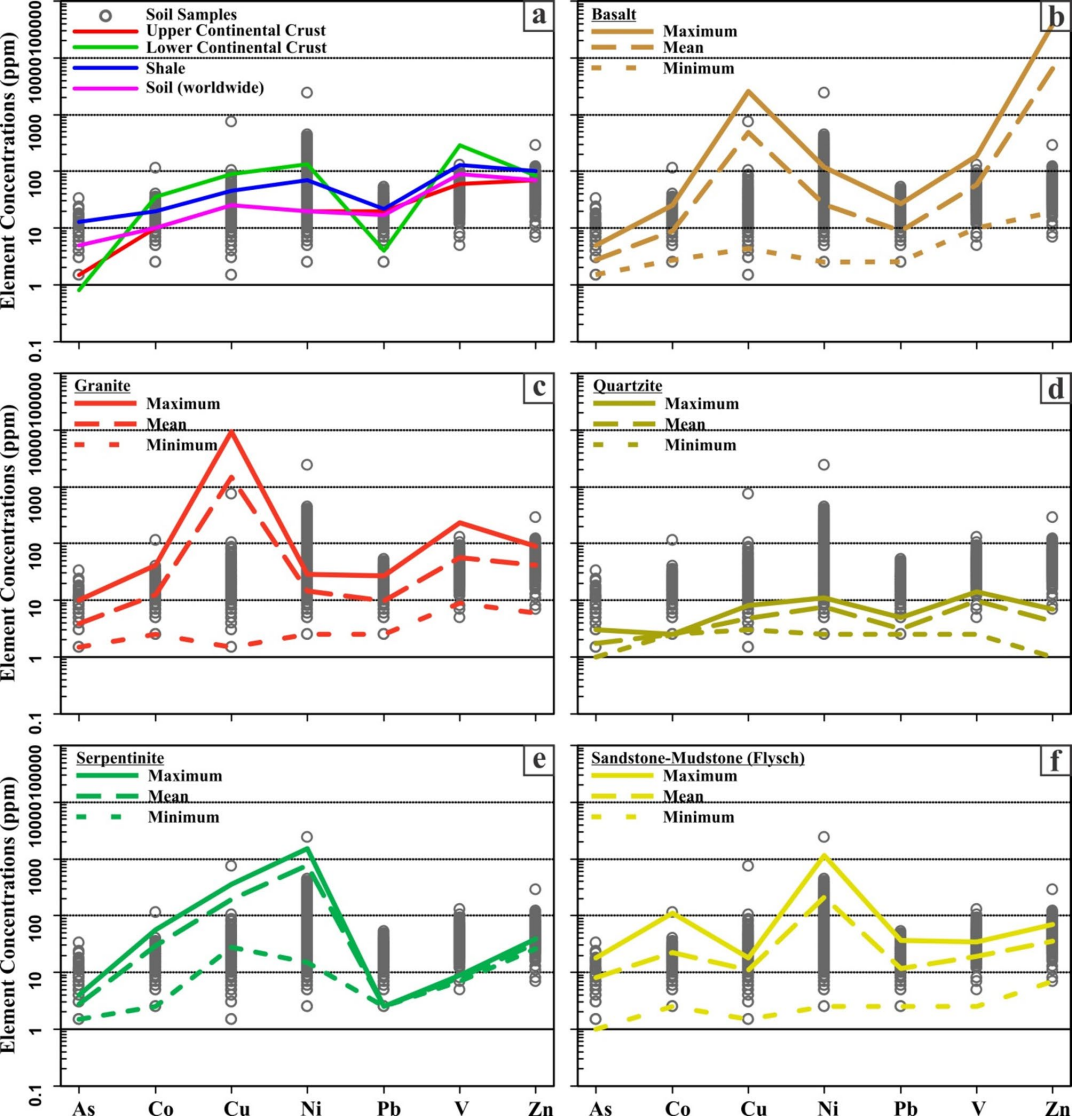
The content of HMs in the soil samples are compared to the values of: (a) the upper and lower continental crust, shale and soil (worldwide), (b) basalt, (c) granite, (d) quarzite, (e) serpentinite, and (f) sandstone-mudstone (flysch) in the study area.

The concentrations of HMs in the soil samples were compared to those of the upper and lower continental crust, shale, and soil (worldwide), as well as various local rocks found in the study area, including basalt, granite, quartzite, serpentinite, and sandstone/mudstone (flysch formation). Table 3; Fig. 4a show that some soil samples have higher levels of all HMs (except vanadium) compared to the mean values of the upper and lower continental crust, shale, and soil (worldwide). Additionally, some soil samples exhibit an enrichment of vanadium compared to the mean values of the upper continental crust and soil (worldwide). These enrichments potentially point out the sources of soil pollution.

Arsenic levels in the area differ depending on the local type of rocks, with basalt ranging from 1.5 to 5 ppm, granite ranging from 1.5 to 10 ppm, serpentinite ranging from 1.5 to 4 ppm, and flysch formations ranging from 1.5 to 18 ppm (Table 3). The majority of soil samples exhibit higher levels of arsenic compared to the maximum values found in the samples of basalt, granite, quartzite, and serpentinite (Fig. 4b, c, d and e). However, they mostly exhibit lower levels of arsenic in comparison to those observed in flysch formations (Fig. 4f). The maximum cobalt contents in the local rocks are 25 ppm in basalt, 42 ppm in granites, 56 ppm in serpentinites, and 112 ppm in the flysch formations (Table 3). Soil samples, except for sample 2, exhibit cobalt levels lower than those found in the samples of granite, serpentinite, and flysch formations. Most soil samples also have cobalt levels lower than the maximum value observed in basalt (Fig. 4b, c, d and e). Copper contents within the local rocks vary from 4 to 2601 ppm for basalt, 1.5 to 9416 ppm for granite, 1.5 to 8 ppm for quartzite, 28 to 363 ppm for serpentinite, and 1.5 to 18 ppm for the flysch formations (Table 3). It is noteworthy that all soil samples have relatively low levels when compared to the maximum values obtained for basalt, granite, and serpentinite. Nickel contents within the study area exhibit ranges of 2.5 to 118 ppm for basalt, 2.5 to 29 ppm for granite, 2.5 to 11 ppm for quartzite, 15 to 1532 ppm for serpentinite, and 2.5 to 1146 ppm for the flysch formations (Table 3). The soil samples mostly displayed higher nickel levels than basalt, granite, and quartzite (Fig. 4b and d). However, the nickel levels in all soil samples (excluding sample 2) were lower than those found in serpentinite and flysch formations (Fig. 4e and f). The basalt and granite formations have recorded lead contents of 27 ppm at the highest, while the flysch formations have 37 ppm (Table 3). Most of the soil samples exhibit decreased levels of lead compared to the maximum levels detected in basalt, granite, and flysch formations (Fig. 4b, c, and f). On the contrary, soil samples show an enrichment relative to the maximum lead values of quartzite and serpentinite (Fig. 4d). The contents of vanadium between various local rock types show significant differences. Basalt has a range of vanadium content from 10 to 187 ppm, while granite has a content that ranges from 9 to 230 ppm. Quartzite has a range of 2.5 to 14 ppm, serpentinite ranges from 7 to 9 ppm, and the flysch formations have a range of 2.5 to 34 ppm (Table 3). All the soil samples have exhibited lower vanadium contents compared to the maximum values found in basalt and granite (Fig. 4b and c). Zinc contents vary greatly, ranging from 19 to 38,246 ppm in basalt, 6 to 89 ppm in granite, 1 to 7 ppm in quartzite, 26 to 39 ppm in serpentinite, and 7 to 69 ppm in the flysch formations (Table 3).

**Table 3:** The descriptive values of the upper and lower continental crust, shale and soil (worldwide) and various local rock formations found in the study area.

		As	Co	Cu	Ni	Pb	V	Zn	References
		ppm							
Upper Continental Crust	Mean	1.5	10.0	25.0	20.0	20.0	60	71	^67^
Lower Continental Crust	Mean	0.8	35.0	90.0	135.0	4.0	285	83	
Shale	Mean	13.0	20.0	45.0	70.0	22.0	130	100	^64,65^
Soil (worldwide)	Mean	5.0	10.0	25.0	20.0	17.0	90	70	
Basalt	Minimum	1.5	2.5	4.0	2.5	2.5	10.0	19.0	This Study
	Maximum	5.0	25.0	2601.0	118.0	27.0	187.0	38246.0	
	Mean	2.5	9.0	494.7	26.3	9.0	58.5	6414.7	
	N	6							
Granite	Minimum	1.5	2.5	1.5	2.5	2.5	9.0	6.0	
	Maximum	10.0	42.0	9416.0	29.0	27.0	230.0	89.0	
	Mean	3.6	12.4	1456.0	14.5	9.6	56.7	41.1	
	N	12							
Quartzite	Minimum	1.5	2.5	1,5	2.5	2.5	2.5	1.0	
	Maximum	1.5	2.5	8.0	11.0	2.5	14.0	7.0	
	Mean	1.5	2.5	4.4	7.6	2.5	9.6	4.3	
	N	4							
Serpentinite	Minimum	1.5	2.5	28	15	2.5	7	26	
	Maximum	4	56	363	1532	2.5	9	39	
	Mean	2.8	29.3	195.5	773.5	2.5	8	32.5	
	N	2							
Sandstone and mudstone (Flysch)	Minimum	1.5	2.5	1.5	2.5	2.5	2.5	7.0	
	Maximum	18.0	112.0	18.0	1146.0	37.0	34.0	69.0	
	Mean	8.1	22.4	11.1	211.5	11.7	18.9	35.0	
	N	6							

### Risk assessment of HMs

The statistical results of the four calculated integrated pollution indexes are presented in Table 4. The results of index calculations in the soil samples vary between 0.7 and 41 for C_d_, 0.1 and 5.9 for mC_d_, 0.0002 and 1.8 for ESPI, and 0.1 and 1.6 for PLI (Table 4).

**Table 4:** Descriptive statistics for the C_d_, mC_d_, ESPI, and PLI indexes.

Descriptive Value	C_d_	mC_d_	ESPI	PLI
Minimum	0.7	0.1	0.0002	0.1
Maximum	41.0	5.9	1.8	1.6
Mean	3.9	0.6	0.01	0.4
Standard deviation	2.4	0.3	0.08	0.2
Skewness	7.0	7.0	19.8	0.9
Kurtosis	93.8	93.6	410.0	1.9
N	719			

**Fig. 5:**
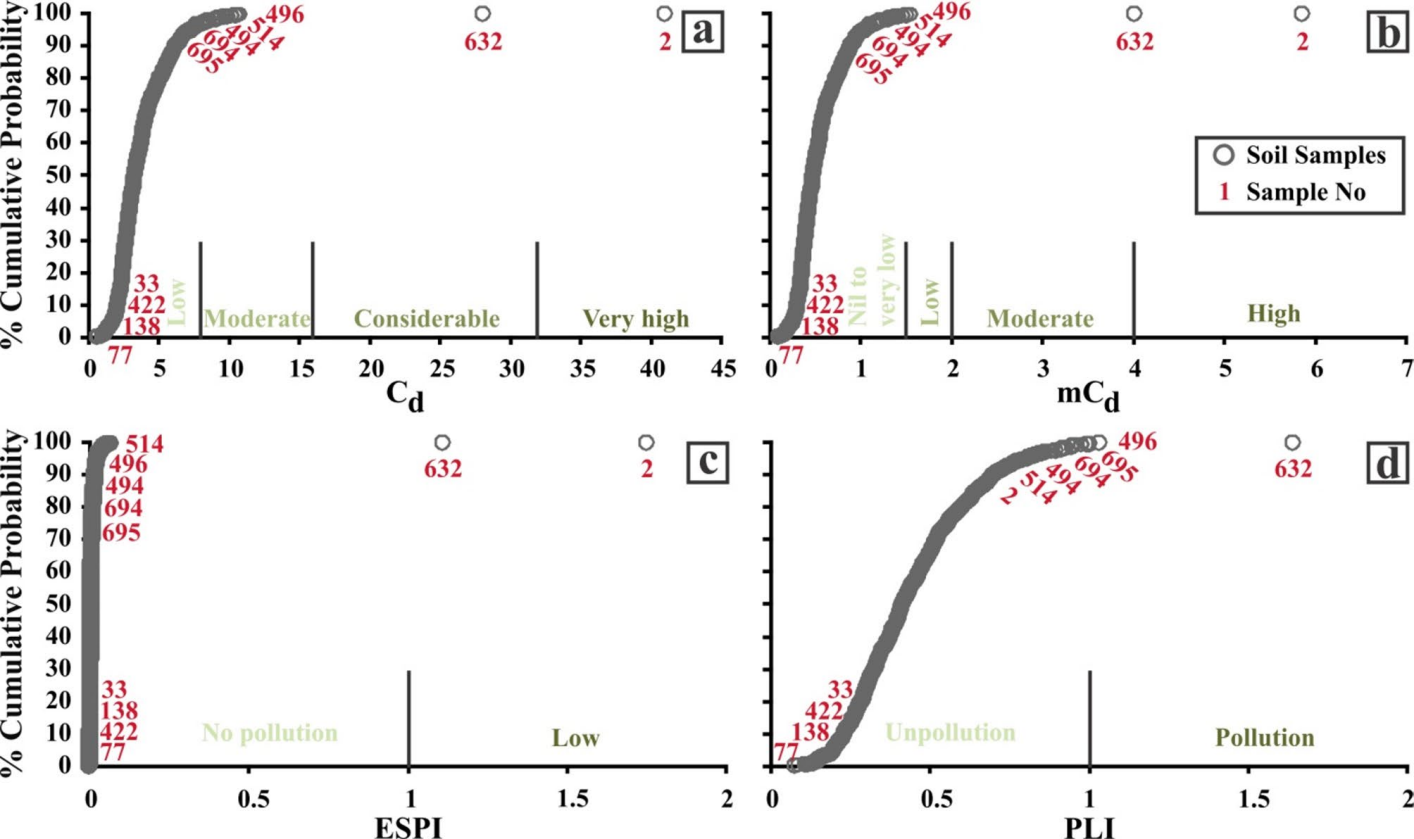
The cumulative probability (%) plots for the C_d_ (a), mC_d_ (b), ESPI (c), and PLI (d).

The results for C_d_ indicate that twenty-four soil samples have a moderate to very high degree of contamination (Fig. 5a). The assessment shows that sample 2 has a very high degree of contamination, while sample 632 shows a considerable degree of contamination. Twenty-two soil samples (including samples 496, 514, 494, 694, and 695) have C_d_ values ranging from 8 to 16, expressing a moderate degree of contamination. The remaining samples, which account for 96.7% of the total, exhibit a low degree of contamination attributed to C_d_ as given in Fig. 5a. The mC_d_ results show that five soil samples (samples 2, 632, 496, 514, and 494) have varying degrees of contamination between low and high. The mC_d_ values of samples 2 and 632 suggest high levels of contamination, whereas samples 496, 514, and 494 suggest low levels of contamination (Fig. 5b). The remaining 99.3% of soil samples indicate nil to very low contamination based on the mC_d_. In addition, the mC_d_ index scores were observed to be similar in the order to the C_d_ index scores for each soil sample. However, the narrow category class ranges in mC_d_ resulted in fewer samples that could potentially have contamination compared to the C_d_ index. An obvious and statistically significant positive correlation was observed at a confidence level of 99% between the C_d_ and mC_d_ due to their analogous calculation processes (Table 5). The calculation of the ESPI index demonstrates that only two samples present a low risk of pollution. According to the ESPI index scores, samples 2 and 632 pose a low pollution risk, while the other samples pose no risk of pollution, accounting for 99.7% of the total (Fig. 5c). In addition, the rankings of ESPI index scores for each soil sample are largely consistent with those of C_d_ and mC_d_, which is also supported by the high positive correlation (*r* = 0.892 and 0.893) between the three indexes (Table 5). The PLI index results reveal that four soil samples have a PLI value surpassing 1, signifying a potential risk of pollution. Based on the scores from the PLI index, the soil samples 632, 496, 695, and 694 illustrate a pollution risk, while the remaining samples, accounting for 99.4% of the total, do not pose any pollution risk (Fig. 5d). Table 5 indicates a significant positive correlation above the level of 84.4% between C_d_, mC_d_, ESPI, and PLI outcomes, pointing towards their calculations being mostly consistent.

**Table 5:** Spearman’s Rho correlation matrix between integrated pollution indexes.

	C_d_	mC_d_	ESPI	PLI
C_d_	1.000			
mC_d_	1.000^**^	1.000		
ESPI	0.892^**^	0.893^**^	1.000	
PLI	0.968^**^	0.968^**^	0.844^**^	1.000

** Correlation is significant at the 0.01 level (2-tailed).

### Spatial distribution maps of integrated pollution indexes

**Fig. 6:**
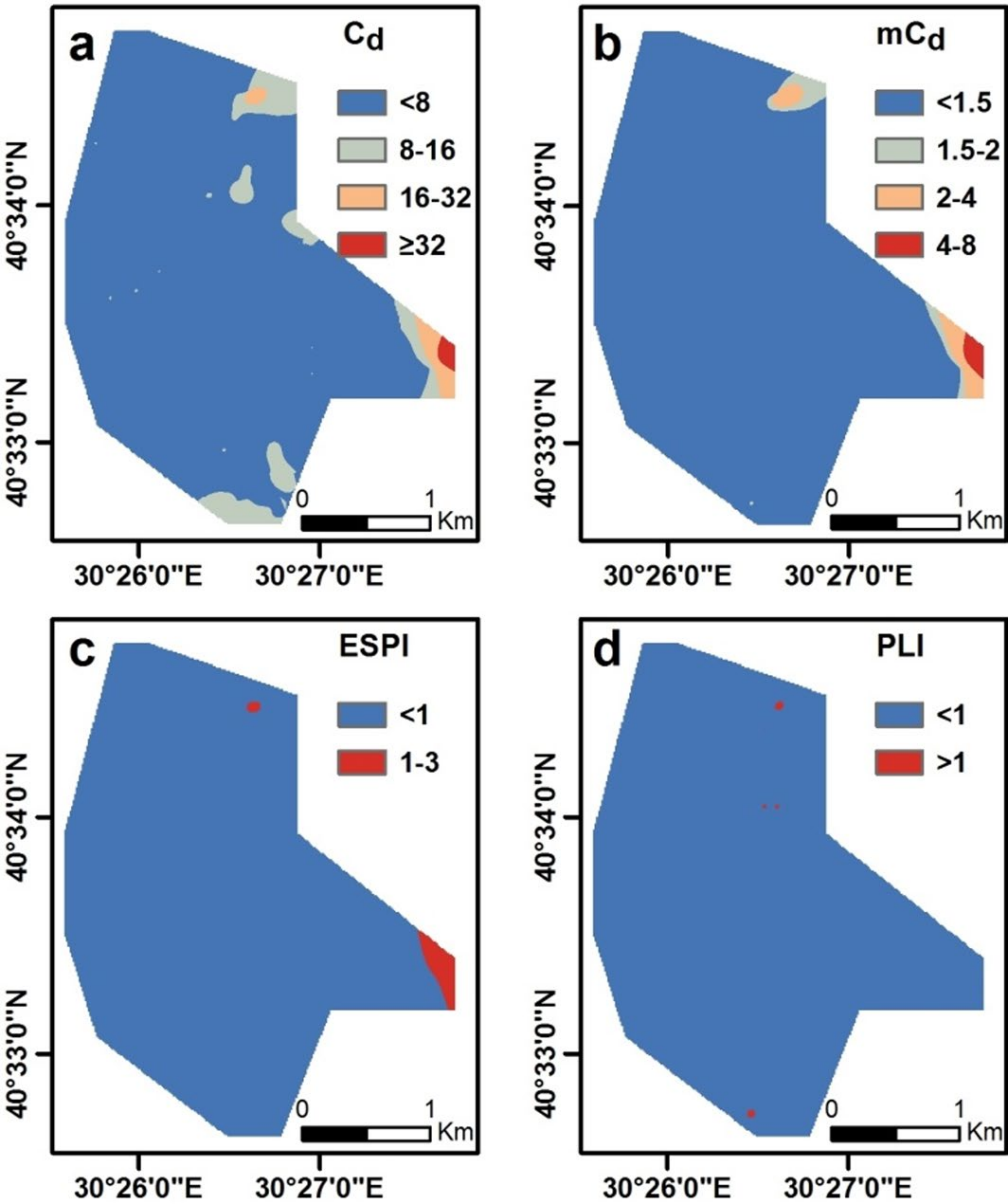
Spatial distribution maps of the C_d_ (a), mC_d_ (b), ESPI (c), and PLI (d).

The spatial distribution of C_d_ shows high values in the northeast, central-east, southeast, and southwest of the study area and is largely consistent with the mC_d_ contamination pattern (Fig. 6a and b). The ESPI values are found to be concentrated in the northeast and southeast sectors of the study area and mostly correspond to similar areas of the C_d_ and mC_d_. However, it is noteworthy that the ESPI occupies a smaller area than the C_d_ and mC_d_ (Fig. 6c). The reason for this is that the ESPI identified fewer samples with soil contamination potential. The PLI index calculations are concentrated in the study area’s northeast and southwest, mostly consistent with the distribution patterns of other integrated indexes (Fig. 6d).

### Multivariate statistical analyses

The correlation coefficients between the contents of HMs in the soil samples are presented in Table 6. Arsenic exhibits the highest positive correlation with lead, followed by zinc, vanadium, copper, cobalt, and nickel in decreasing order. In contrast, there is a significant positive correlation between cobalt, copper, and nickel, which is more than 77.4% (Table 6). Zinc has a positive correlation with copper at a level of 0.675 (r).

**Fig. 7:**
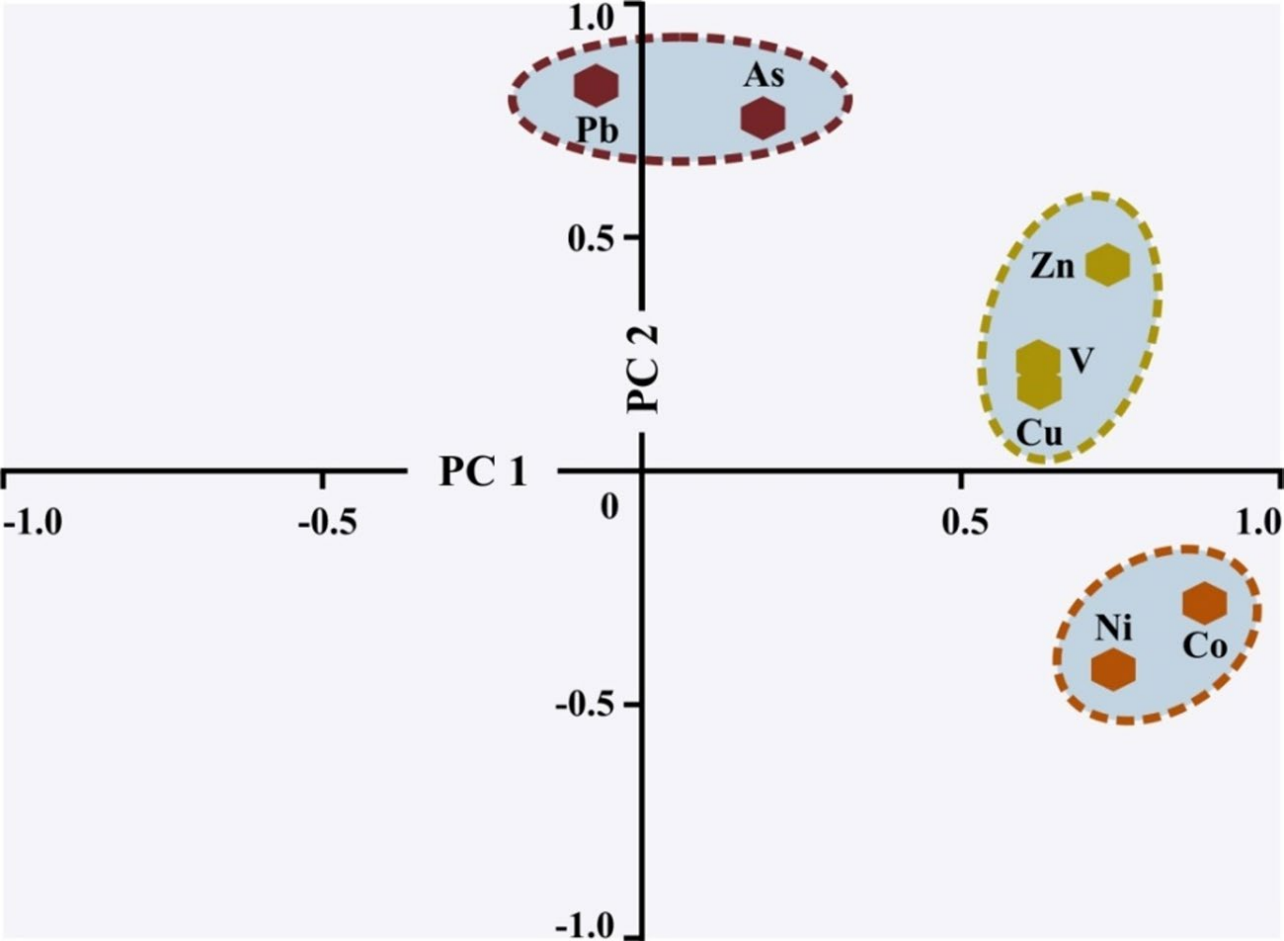
The binary diagram for two PCs (PC1 and PC2).

The results of the PCA illustrate that two PCs account for 63.7% of the total variance (Table 7). The first component (PC1) represents 38.1% of the total variance and displays positive loading of cobalt (0.88), nickel (0.74), zinc (0.73), copper (0.62), and vanadium (0.62). The second principal component (PC2) accounts for 25.6% of the total variance. It is primarily controlled by lead (0.82) and arsenic (0.75). The binary diagram (Fig. 7) suggests that the presence of three distinct groups consists of arsenic-lead, copper-zinc-vanadium, and cobalt-nickel.

**Fig. 8:**
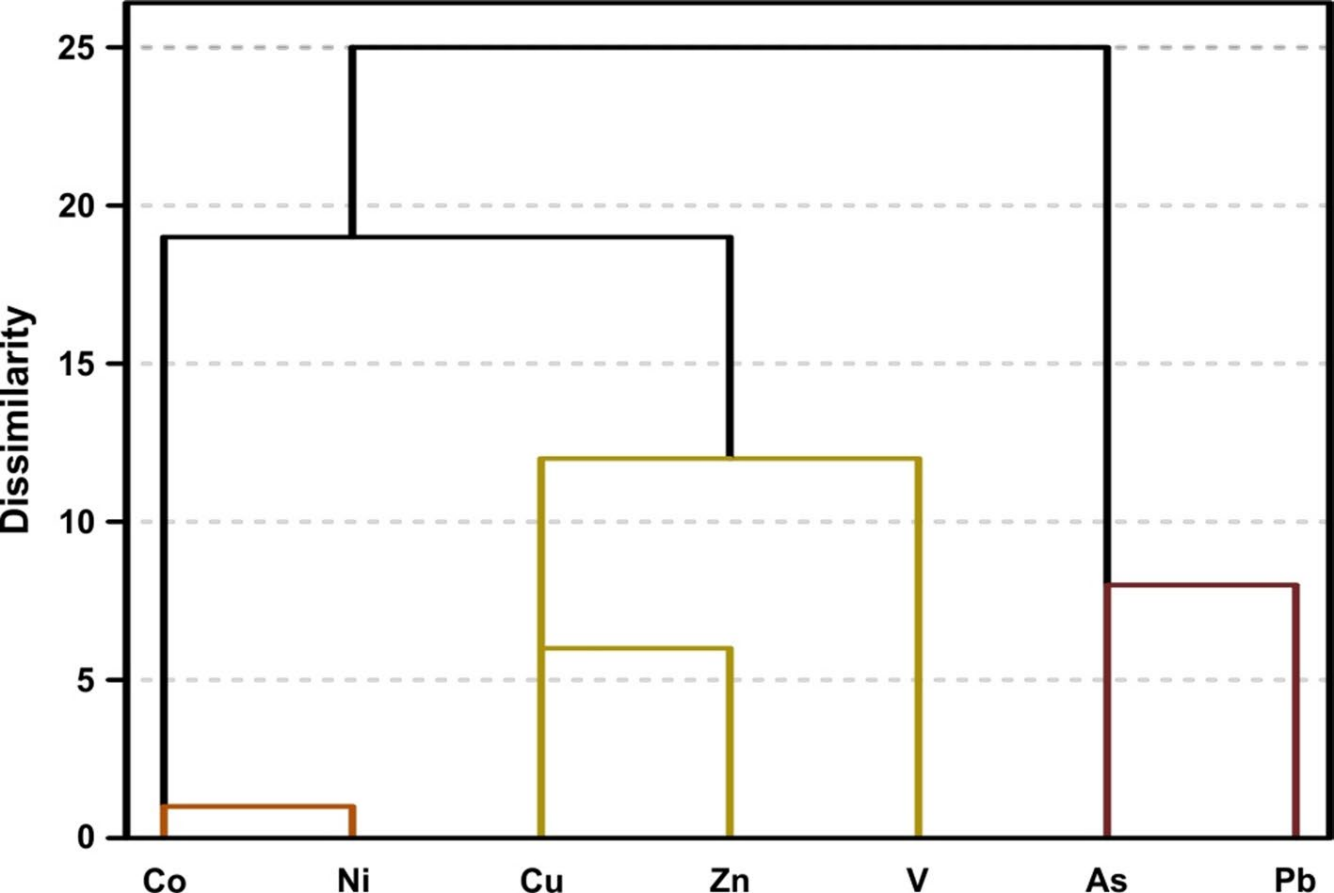
The dendrogram for the HMs contents.

The dendrogram in Fig. 8 depicts the results of R-mode HCA for HMs contents. The analysis identifies two main clusters that are statistically significant. The first main cluster is divided into subcluster-1, containing cobalt and nickel, and subcluster-2, containing copper, zinc, and vanadium. The second main cluster comprises arsenic and lead. The results of HCA are consistent with correlation analysis and PCA.

**Table 6:** Spearman’s Rho correlation matrix for the concentrations of HMs.

Parameters	As	Co	Cu	Ni	Pb	V	Zn
As	1						
Co	0.100^**^	1					
Cu	0.210^**^	0.851^**^	1				
Ni	0.009	0.873^**^	0.774^**^	1			
Pb	0.493^**^	− 0.226^**^	-0.051	− 0.377^**^	1		
V	0.247^**^	0.589^**^	0.583^**^	0.418^**^	0.100^**^	1	
Zn	0.329^**^	0.612^**^	0.675^**^	0.415^**^	0.214^**^	0.576^**^	1

** Correlation is significant at the 0.01 level (2-tailed).

**Table 7:** Loading matrix of the PCs and the total variance explained by each PC.

Parameters	PCs	
	PC1	PC2
Co	**0.88**	-0.28
Ni	**0.74**	-0.43
Zn	**0.73**	0.44
Cu	**0.62**	0.18
V	**0.62**	0.23
Pb	-0.07	**0.82**
As	0.19	**0.75**
Eigenvalues	2.68	1.78
% of variance	38.1	25.6
Cumulative %	38.1	63.7

Significant values are in bold.

### Vertical distribution of HMs

**Fig. 9:**
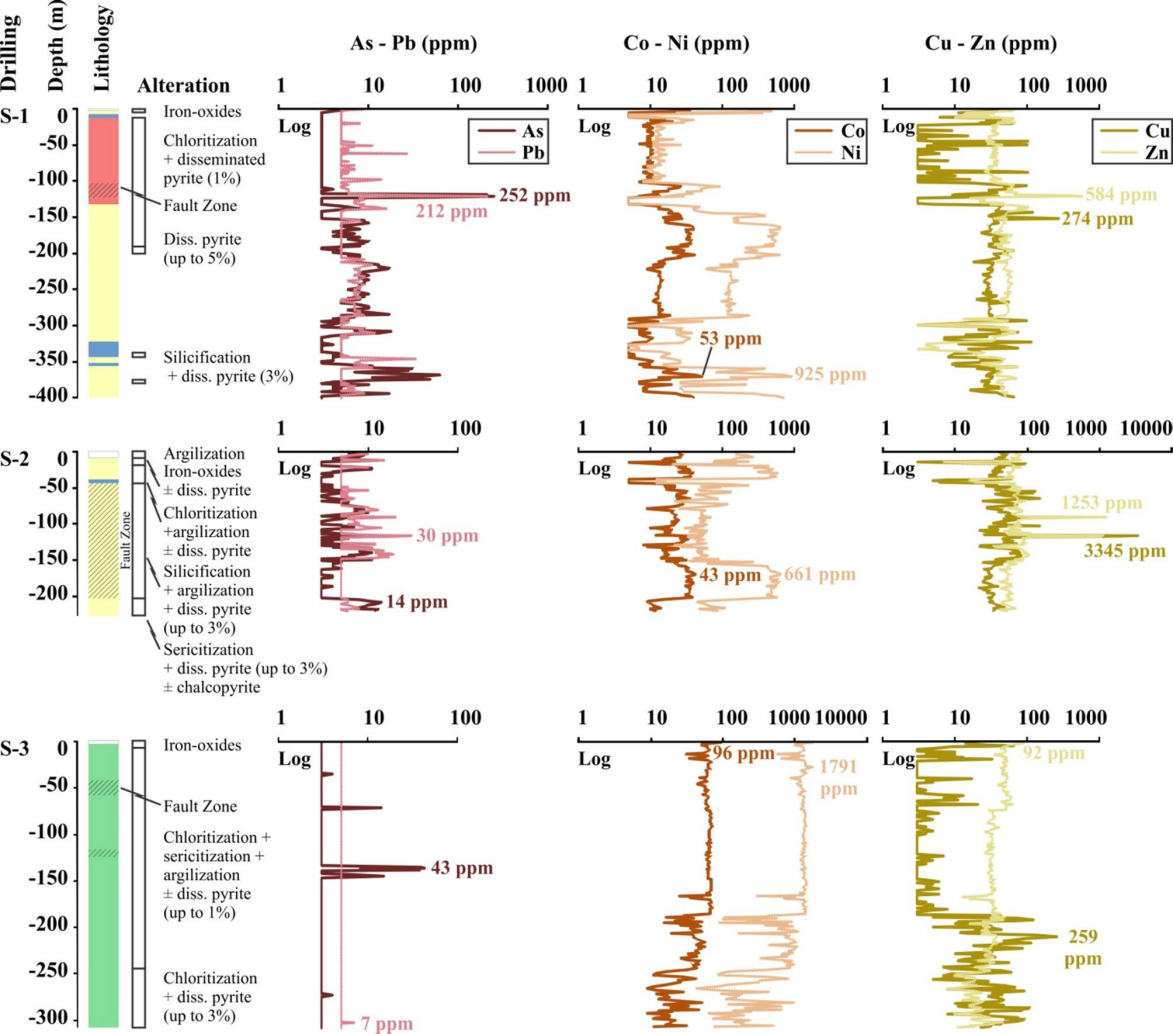
Lithochemical logging was undertaken on drilling S-1, S-2, and S-3 to ascertain the vertical distribution pattern of As-Pb, Co-Ni, and Cu-Zn (See Fig. 2 for explanations of the colours used to describe lithology. The fault zone is indicated by oblique lines.).

The geochemical results of drillcore samples from the drilling S-1 exhibit a wide range of values for arsenic (1.5 to 252 ppm), cobalt (2.5 to 53 ppm), copper (1.5 to 274 ppm), nickel (2.5 to 925 ppm), lead (2.5 to 212 ppm), and zinc (1.5 to 584 ppm) (Table 8). The highest contents of arsenic, copper, lead, and zinc were enriched relative to those in the upper continental crust by 168, 11, 10, and 8 times, respectively. The given values correspond to the levels of disseminated pyrite, which can reach up to 1% in volume, and chloritization, along the fault zone that has developed within the granites (Fig. 9). At greater depths, silicification increases, and disseminated pyrite has reached up to 5% in volume. However, this variation did not have a significant impact on the contents of HMs. The highest enrichments of cobalt and nickel coincide with the flysch formation levels. At these levels, cobalt and nickel demonstrate a synchronous increasing and decreasing pattern, regardless of the other HMs (Fig. 9).

The drillcore samples from the drilling S-2 contain between 1.5 and 14 ppm of arsenic, 2.5 and 43 ppm of cobalt, 1.5 and 3345 ppm of copper, 12 and 661 ppm of nickel, 2.5 and 30 ppm of lead, and 7 and 1253 ppm of zinc (Table 8). The highest levels of arsenic, copper, lead, and zinc were enriched relative to the upper continental crust by 9, 134, 1.5, and 18 times, respectively. Similar to those in drilling S-1, the highest values correspond to the levels of silicification, argilization, and disseminated pyrite (up to 3% in volume) ± chalcopyrite along the fault zone. The negative distribution patterns between the contents of cobalt-nickel and copper-zinc in the drilling S-2 are quite evident (Fig. 9).

The geochemical results of the drillcore samples from the drilling S-3 show varying levels of arsenic (1.5–43 ppm), cobalt (9–96 ppm), copper (1.5–259 ppm), nickel (43-1791 ppm), lead (2.5-7 ppm), and zinc (8–92 ppm) (Table 8). The levels of arsenic, cobalt, copper, and nickel are significantly higher (29, 10, 10, and 90 times, respectively) than the typical values found in the upper continental crust. The drilling S-3 features high levels of cobalt and nickel, consistent with the levels found in serpentinites. These levels match the lithochemical distribution patterns observed in the other two drillings. It is worth noting that the cobalt and nickel contents decrease significantly after 200 m (Fig. 9).

**Table 8:** Descriptive statistics for the geochemical results of the drillcore samples.

Drilling No	Depth	Descriptive value	As	Co	Cu	Ni	Pb	Zn
	meter		ppm	ppm	ppm	ppm	ppm	ppm
S-1	401	Minimum	1.5	2.5	1.5	2.5	2.5	1.0
		Maximum	252.0	53.0	274.0	925.0	212.0	584.0
		Mean	7.7	13.6	25.1	107.2	5.8	45.5
		Standard deviation	24.8	9.2	26.9	165.9	13.5	44.1
		Skewness	8.2	1.6	3.9	2.3	14.2	9.7
		Kurtosis	71.0	2.5	29.2	4.8	217.5	108.4
		N	256					
S-2	221	Minimum	1.5	2.5	1.5	12.0	2.5	7.0
		Maximum	14.0	43.0	3345.0	661.0	30.0	1253.0
		Mean	4.7	22.9	70.8	175.1	5.5	86.7
		Standard deviation	3.3	9.5	271.7	193.1	4.7	150.3
		Skewness	0.8	-0.1	11.5	1.2	2.0	7.0
		Kurtosis	-0.2	-0.6	137.1	-0.2	4.8	48.6
		N	158					
S-3	309	Minimum	1.5	9.0	1.5	43.0	2.5	8.0
		Maximum	43.0	96.0	259.0	1791.0	7.0	92.0
		Mean	2.2	50.2	18.4	980.3	2.52	34.3
		Standard deviation	4.5	19.0	30.2	469.2	0.3	12.2
		Skewness	7.9	-0.8	3.4	-0.8	15.4	0.6
		Kurtosis	64.2	-0.7	18.3	-0.8	237.0	1.4
		N	237					

## Discussion

The analysis results of 719 soil samples from the study area revealed that 3.3%, 0.7%, 0.3%, and 0.6% of the soil samples were contaminated with HMs on the basis of the index calculations of C_d_, mC_d_, ESPI, and PLI, respectively. Although these values suggest relatively low levels of HMs pollution across the entire study area, they do not reflect the elevated concentrations of HMs detected in specific local areas. The common intersection areas with the highest levels of pollution, according to the four index scores, are located in the northeast and southeast parts of the study area. In addition, relatively lower levels of pollution are observed in the central-eastern and southwestern regions (Fig. 6). It is possible that different or similar pollution sources exist in these areas, which were exposed to pollution and monitored independently at two main distinct locations. The results of the PCA and HCA, along with the identified positive and negative correlations between HMs, suggest the presence of various assemblages, including cobalt-nickel, copper-zinc-vanadium, and arsenic-lead. The key implication of these findings is that assemblages of HMs may exhibit co-occurrence in the soil or share a common natural and/or anthropogenic sources.

Compared to the shale and soil (worldwide) mean values, cobalt exhibits a 6-times and a 11-times enrichment, respectively, while nickel exhibits a 34-times and a 119-times enrichment, respectively (Tables 2 and 3). In addition, the concentrations of cobalt and nickel in the soils are 2–5 and 3-119 times higher, respectively, than those reported in previous studies on HM levels in soils and sediments around the study area (Table 9)^14,31,41–43,58^. As a result of these increases, areas of high contamination have developed in this part of the study area (Fig. 6). Lithogeochemical relationships demonstrate that cobalt and nickel exert a significant influence on their variations, particularly in areas distant from agricultural activities in forested areas, which represent the majority of the soil samples. However, with the exception of sections containing serpentinite blocks, the flysch formation, which consists of sandstone, siltstone, mudstone, and older fragments and blocks, is unlikely to be the primary geogenic source responsible for the enrichment of these HMs. More importantly, lithogeochemical analyses of drillcore and surface rock samples reveal that serpentinites are the rock lithologies with the highest cobalt and nickel contents (Tables 3 and 8). The high nickel (up to 220 ppm) and chromium (up to 173 ppm) contents detected in soils close to the southwestern border of the study area support the research findings (Table 9)^41,42^. Similarly, high cobalt (up to 51 ppm) and nickel (up to 750 ppm) contents have been reported in sediments taken from stream systems passing through serpentinites and flysch formations northeast of Geyve^58^.

**Table 9:** A comparative data of the concentration of HMs in soils and sediments around the Sakarya-Geyve region.

Location	Sample Type	As	Co	Cu	Ni	Pb	V	Zn	References
		ppm	ppm	ppm	ppm	ppm	ppm	ppm	
South of Lake Sapanca, Kınalı-Sakarya Highway	Soil	-	-	-	0-58.67	0-47.54	-	-	^43^
Lake Sapanca	Sediment	-	-	17.28–35.51	20.10-34.31	12.94–17.80	-	39.02–75.31	^14^
North of Lake Sapanca, D-100 Highway	Soil	-	21.92	69.42	47.70	227.60	20.72	229.07	^31^
North-Northeast of Geyve	Soil	3.24–6.62	-	24.52-108.18	89.06-219.88	3.95–21.10	-	54.95-149.58	^41,42^
Northeast of Geyve	Sediment	1.5-9	2.5–51	9-106	10–750	2.5–37	9–61	5-170	^58^
Northeast of Geyve	Soil	1.5–33	2.5–114	1.5–746	2.5–2384	2.5–53	2.5–128	7-290	This Study

(-) : Not available data.

The coexistence of metals depends on the similarity of their ion radii and chemical properties. Some metals have very similar ion radii and chemical properties (e.g., cobalt-nickel and molybdenum-tungsten). Although the chemical properties of metals that are only close to each other in their ion radii are quite different from each other, they can often coexist (e.g., nickel–magnesium and lead-zinc)^68^. Nickel and cobalt, as moderately siderophile elements, have similar ion radii, share similar geochemical characteristics, and exhibit comparable behavior in magmatic processes^69–72^. During the crystallization of ultramafic magmas, they tend to be enriched in the sulfide phase. In particular, under atmospheric conditions, the weathering of ultramafic rocks results in the partial mobilization of nickel, leading to its transformation into magnesium-nickel silicates, whereas cobalt preferentially binds to iron-aluminum-manganese oxides^73^. Cobalt and nickel may gradually become enriched in ultramafic rocks and their associated soils as a result of surface alteration processes, as well as increasing degrees of weathering and dissolution^74^. The similar behavior between cobalt and nickel in the soils of the study area is confirmed by the correlation matrix at the 0.873 level (Table 6). Both HMs predominantly exhibited consistent increasing and decreasing patterns throughout all drillings (Fig. 9).

Compared to the shale mean values, copper exhibits a 17-times enrichment, zinc a 43-times enrichment, and vanadium a 0.98-times enrichment (Tables 2 and 3). When compared to soil (worldwide) mean values, copper exhibits a 30-times enrichment, zinc a 4-times enrichment, and vanadium a 1.4-times enrichment (Tables 2 and 3). Moreover, the concentrations of copper, zinc, and vanadium are significantly higher than those reported in previous studies on HMs levels in soils and sediments around the study area (Table 9)^14,31,41,42,58^. The correlation matrix results indicate a positive anomaly with a minimum value of 0.576 among the three HMs (Table 6). This finding is further supported by the spatial distribution maps of their concentrations and the relationships between the index scores (Figs. 3 and 6). Lateral and vertical geological investigations conducted on rocks in the soil sampling areas revealed the genetic relationships between copper, zinc, and vanadium enrichments and the rock lithologies to which the soils are temporally and spatially linked. In the study area, vanadium concentrations are highest in basalts and granites, exhibiting a 3-times and a 4-times enrichment, respectively, compared with those in the upper continental crust (Table 3). Copper concentrations in basalt and granite are 104 and 376 times higher, respectively, than the typical copper content of the upper continental crust. Similarly, the highest zinc concentration was observed in basalt, representing a 538-times enrichment compared to the average zinc value of the upper continental crust. Geochemical analysis of drillcore samples collected from the flysch formation and fault and alteration zones in the S-1 and S-2 drillings (Fig. 9) reveals that copper and zinc are enriched a 134-times and a 18-times, respectively, compared to the upper continental crust, which is consistent with the geochemical data from surface rock samples. Furthermore, significant enrichment of copper and zinc relative to the upper continental crust is observed at points corresponding to fault and alteration zones in the all drillings. Both HMs exhibit consistent patterns of increase and decrease throughout the drillcores (Fig. 9).

Compared to the shale and soil (worldwide) mean values, arsenic exhibits a 3-times and a 7-times enrichment, respectively, whereas lead exhibits a 24-times and a 3-times enrichment, respectively (Tables 2 and 3). Furthermore, the concentrations of arsenic and lead are higher than those reported in previous studies on HMs levels in soils and sediments around the study area^14,41–43,58^, with the exception of the D-100 highway in northern of Lake Sapanca (Table 9)^31^. The correlation matrix results indicate a positive anomaly of 0.493 between these two HMs (Table 6), further supported by the spatial distribution maps of their HMs concentrations and the relationships between their index scores (Figs. 3 and 6). In surface rock samples, arsenic concentrations are 3 times higher in basalts, 6 times higher in granites, and 12 times higher in flysch compared to the upper continental crust (Table 3). In core samples collected from the S-1 drilling, corresponding to granitic rocks and fault zones, arsenic is enriched a 168-times, and lead is enriched a 10-times relative to the upper continental crust (Fig. 9). Additionally, arsenic and lead concentrations slightly increase at points corresponding to faults and alteration zones in all drillings. Both elements exhibit similar distribution patterns throughout the drillings (Fig. 9).

Considering hydrothermal processes, copper, zinc, vanadium, lead, and arsenic may be present in certain types of mineral deposits (e.g., porphyry copper-gold, carbonate-hosted lead-zinc-copper, and intermediate epithermal gold-silver deposits) within the ore and/or alteration zones. In such systems, vanadium is released as a result of hydrothermal alteration processes, primarily through the dissolution of ferromagnesian minerals such as amphibole and biotite in granitic host rocks, and olivine and pyroxene in basaltic host rocks^74^. Copper, zinc, lead, and arsenic may be partially or completely enriched together as a result of the interaction between hydrothermal fluids rich in these metals and the host rocks. These reaction zones, characterized by mineralizations and alterations in the rocks, are typically associated with fault zones. In the examined drillcore samples, particularly within fault zones, widespread alteration products such as pyritization, chloritization, silicification, and argillization are observed (Fig. 9). Fault zones act as major pathways for hydrothermal fluid flow and HM mobilization, particularly under supergene oxidation conditions. NE-SW trending faults, identified through geological mapping and drillcore analyses, facilitate fluid circulation, chemical weathering, and secondary HM enrichment. Elevated HM concentrations in soils are closely associated with fault-controlled zones of mineralization and alteration, further highlighting the structural control on supergene processes (Figs. 2 and 9).

Geochemical comparisons of the soil and rock (surface and drillcore) samples suggest that elements such as vanadium, copper, and zinc, along with lead and arsenic, tend to exhibit simultaneous enrichment. Therefore, it is understood that the weathering and erosion of potential subsurface ore deposits and associated rocks, resulting from atmospheric weathering and hydrothermal processes, can lead to the enrichment of these HMs in the soils of these areas^75^. The copper concentrations detected in areas close to the sampling site, reaching up to 0.52% (Fig. 2b)^45^, along with the anomalous copper and zinc enrichments identified in surface rock and drillcore samples from the study area, suggest a potential genetic link between the HMs content in soils of specific regions and the underlying mineral deposits (Tables 3 and 8; Fig. 9). Subsurface polymetallic mineral deposits may undergo partial or complete oxidation due to water circulation along fault zones^76^. Primary (hypogene) sulfide minerals (e.g., galena, sphalerite, chalcopyrite, and pyrite) are unstable in the oxidation zone and decompose much faster than the associated gangue and silicate minerals. During this decomposition, sulfide minerals transform into secondary (supergene) sulfate minerals. While those with low solubility precipitate in situ (e.g., PbSO_4_), highly soluble species (e.g., Fe^2+^, Cu^2+^, and Zn^2+^) dissolve in water and dissociate into ions. Among the released metal ions, those with low mobility (e.g., Fe^2+^) react with H_2_O and O_2_ in the environment, forming hydroxide and oxide mineral phases. In contrast, highly mobile metals such as Cu^2+^ and Zn^2+^ remain dissolved and become enriched near the groundwater level. These metals precipitate as hydroxides, oxides, sulfates, carbonates, or native metals in the upper part of the groundwater level, whereas in the lower part, they precipitate as secondary sulfide and sulfate minerals^77^. Under such supergene alteration conditions, the oxidation of arsenic-rich pyrites can lead to the decomposition of pyrite and the subsequent release of arsenic into the environment, resulting in arsenic contamination of soils^78^. In this context, it has been reported that the enrichments of copper, arsenic, zinc, and lead in the soils of Hezhang County (southwestern China) are attributed to the influence of surface and groundwater interacting with iron and lead-zinc mineral deposits in the region^78^. Isleyen et al.^41^ partially attributed the high arsenic concentrations in the soils of the northern Geyve region to mining activities conducted on gold-bearing quartz veins, which contain pyrite and arsenopyrite. Terzi et al.^58^ and Durgun et al.^79^ associated the lead enrichments determined in the stream sediments of the Geyve region with spatially related ore-bearing granites.

The correlation matrix results revealed positive correlations between cobalt and nickel, as well as among copper, zinc, and vanadium (Table 6). However, unlike cobalt and nickel, the other three HMs are present at low concentrations in serpentinite and flysch formations. Notably, copper, zinc, and vanadium, which are part of PC1, are also present in PC2, despite their low concentrations. These variables are positioned between the arsenic-lead and cobalt-nickel groups in the HCA, indicating that these HMs contribute to both PCs (Table 7; Figs. 7 and 8). This findings suggests that HMs enrichment and contamination are influenced not only by geogenic sources but also by anthropogenic activities, as evidenced by elevated HM levels in soil samples collected near active agricultural areas. Several studies conducted near the study area have reported that the accumulation of HMs in soils is associated with anthropogenic sources, such as vehicle emissions from highways, industrial activities, and urban and agricultural practices in the Geyve region^14,16,31,41–43,58,79–81^. The study area, which is predominantly covered by forest, is situated approximately 800 m above the industrial facilities and urban activities of Geyve and is relatively distant from the highway connecting Ankara and Istanbul. These geographical and environmental factors help minimize the impact of anthropogenic pollutants on most HMs, except in localized cases such as agricultural practices and limited human activities associated with the region’s low population density. In the study area, agricultural practices involving the use of various agrochemical HMs-bearing compounds, such as pesticides and chemical fertilizers^40^, and/or the release of exhaust gases from agricultural vehicles on secondary village roads may contribute to elevated levels of arsenic^82^, cobalt and nickel^42,43,58,72,83,84^, copper^14,42,58,79^, as well as lead and zinc^14,16,31,43,58,79^ in the soil. It is also noteworthy that copper concentrations in the region’s soils may also be influenced by several additional factors, including the feces of animals fed with copper-enriched feeds^42^, domestic waste^14^, and the use of Sakarya River water, which is contaminated with organic and inorganic substances, for agricultural irrigation^41^.

## Conclusions

This study comprehensively evaluated the HMs content and pollution levels of soils in the Geyve region. The concentrations of HMs in some of the soil samples exceeded those of shale, soil (worldwide), upper and lower continental crust, as well as various local rocks found in the study area, indicating that the soils were contaminated with the HMs. The integrated pollution index assessment pointed out that the soils in some parts of the study area were exposed from low to high HMs risk. The results of the correlation analysis revealed a consistent and strong positive correlation among the integrated pollution indexes, with a correlation coefficient exceeding 0.844 (*p* < 0.01). The analyses of geochemical and spatial distributions revealed that high cobalt and nickel concentrations were observed, especially in the southeastern parts where serpentinites were exposed; a strong positive correlation (0.873) between these two HMs was associated with natural geological sources. Such high HMs content of serpentinites supports the effect of rock and soil relationships on geogenic pollution in the region. Furthermore, detailed analyses of geological lithologies identified serpentinites within the flysch formation as the primary source of cobalt and nickel. Copper, zinc, and vanadium were predominantly enriched in the soils, particularly in areas associated with basalt and granite. Drilling studies demonstrated that copper and zinc concentrations were significantly elevated in fault and alteration zones, underscoring the critical role of hydrothermal processes in the enrichment of soils, although anthropogenic sources could not be totally neglected. The correlation (0.493) between arsenic and lead suggests that these HMs are co-distributed through interconnected geological and environmental processes in the region.

The enrichment of HMs in the soils of the Geyve region is influenced by both geogenic and anthropogenic factors. While the impact of anthropogenic pollutants is relatively limited in forested areas, agricultural practices and former mining activities significantly increase HMs accumulation at the local scale. This issue emerges as a critical challenge that must be carefully addressed to ensure both agricultural productivity and environmental sustainability in the region. Therefore, a control program should be established to monitor HMs levels in the soils of the study area continuously while considering local geological characteristics and environmental dynamics.

The continuous increase in the global population has resulted in a growing demand for new agricultural and residential areas, as well as underground resources. Protection of human and environmental health can be achieved primarily through the development of effective land use plans. In this context, it is important to consider the pollution levels and spatial distributions of HMs, which, although economically valuable, pose significant threats to human health because of their toxic effects. This is particularly important for minimizing risks to food safety and the food chain. Furthermore, assessing the contamination levels and distribution patterns of HMs represents a fundamental step in modern soil remediation efforts and guides the selection of appropriate removal methods. However, approaches based on geochemical analysis, elemental distribution data, index calculations, and statistical analyses -primarily conducted within the scope of environmental chemistry and often disregarding local geological features- may sometimes be insufficient to accurately distinguish the origins of HM contaminants in soils. A more accurate identification of HMs sources derived from both geogenic and anthropogenic inputs in the study area is crucial for understanding pollution processes and formulating effective land use strategies. Various isotope techniques (e.g., lead, copper, zinc, iron, and nickel isotopes) are currently employed for understanding metal enrichment processes, explaining ore formation conditions, and classifying the origins of mineral deposits. Notably, isotope data indicative of geogenic sources provide critical insights and valuable geochemical fingerprints for exploring undiscovered subsurface mineralization zones. Similarly, isotope data associated with anthropogenic sources are essential for pollution monitoring, as they offer precise and reliable information on the origins of pollutants. In this context, systematic isotope studies are essential for the study area. In addition to isotope studies, more detailed investigations, including sampling and geochemical analyses (major, trace, and rare earth elements), should be conducted in the areas with high concentrations of HMs in the future. These studies will help to refine the boundaries of polluted areas, confirm the sources of pollution, and increase soil protection against environmental degradation.

In conclusion, this study provides valuable insights into the spatial distribution and potential sources of HMs enrichment in soils across different land-use types, including agricultural and forested areas. The findings highlight the influence of both natural geological background and anthropogenic activities, underscoring the need for site-specific environmental management strategies. By identifying areas of concern, the study offers a scientific basis for informed land-use planning and sustainable agricultural practices. Furthermore, the regional-scale approach adopted here can serve as a model for similar assessments in other agriculturally or geologically sensitive regions. In practice, the data obtained suggest that new agricultural and residential areas planned to be established in the study area should be located away from forested areas that are already enriched in HMs.

## Data Availability

Correspondence and requests for materials should be addressed to G.D. Data will be made available on reasonable request.
